# Distribution, Phytochemical Insights, and Cytotoxic Potential of the *Sesbania* Genus: A Comprehensive Review of *Sesbania grandiflora*, *Sesbania sesban*, and *Sesbania cannabina*

**DOI:** 10.3390/ph18010064

**Published:** 2025-01-09

**Authors:** Fatma Alzahraa Mokhtar, Mariam Ahmed, Aishah Saeed Al Dhanhani, Serag Eldin I. Elbehairi, Mohammad Y. Alfaifi, Ali A. Shati, Amal M. Fakhry

**Affiliations:** 1 Fujairah Research Centre, Sakamkam Road, Fujairah 00000, United Arab Emirates; aishah.suliman@frc.ae; 2 Department of Pharmacognosy, Faculty of Pharmacy, El Saleheya El Gadida University, El Saleheya El Gadida 44813, Egypt; 3 Faculty of Pharmacy and Biotechnology, German University in Cairo, New Cairo 11835, Egypt; mariam.a.aly2003@gmail.com; 4 Biology Department, Faculty of Science, King Khalid University, Abha 9004, Saudi Arabia; serag@kku.edu.sa (S.E.I.E.); alfaifi@kku.edu.sa (M.Y.A.); aaalshati@kku.edu.sa (A.A.S.); 5 Tissue Culture and Cancer Biology Research Laboratory, King Khalid University, Abha 9004, Saudi Arabia; 6 Department of Botany and Microbiology, Faculty of Science, Alexandria University, Alexandria 21511, Egypt; amal.fakhry@alexu.edu.eg

**Keywords:** *Sesbania*, cytotoxicity, galactomannan, nanoparticles, natural cancer treatments, secondary metabolites, cancer pathway inhibition, cytotoxic potential

## Abstract

This review evaluates the cytotoxic potential of the *Sesbania* genus, with a focus on *Sesbania sesban*, *Sesbania grandiflora*, and *Sesbania cannabina*. These species, known for their diverse phytochemical compositions, exhibit notable cytotoxic effects that suggest their utility in natural cancer treatments. Compounds such as quercetin, kaempferol, and sesbagrandiforian A and B have been highlighted for their strong antioxidant and antiproliferative effects, further emphasizing their therapeutic potential. The genus *Sesbania* exhibits a wide range of in vitro and in vivo bioactivities. Extensive research on *S. grandiflora* has uncovered mechanisms such as the activation of caspase cascades and the induction of apoptosis, attributed to its rich content of flavonoids and alkaloids. Notably, sesbanimides derived from *S. grandiflora* seeds have demonstrated potent cytotoxic effects by disrupting mitochondrial function. While *S. sesban* and *S. cannabina* have been less extensively studied, early findings highlight their potential through the inhibition of key cancer pathways and the identification of bioactive compounds such as galactomannan derivatives and 2-arylbenzofurans. Notably, the galactomannan derivatives from *S. sesban* exhibit significant immune-modulating properties. Additionally, nanoparticles synthesized from *Sesbania* species, including Cadmium oxide and PEGylated silver nanoparticles, have demonstrated promising cytotoxic activity by disrupting mitosis and enhancing immune responses. While further research is warranted, the *Sesbania* genus offers a promising basis for the development of innovative anticancer therapies.

## 1. Introduction

Due to its inexpensive cost, easy availability, and minimal or nonexistent adverse effects, herbal medicine is becoming more and more popular in the twenty-first century [1]. It is employed in the treatment of a range of conditions, including irritable bowel syndrome, cancer, allergies, asthma, eczema, premenstrual syndrome, rheumatoid arthritis, fibromyalgia, migraine, menopausal symptoms, and chronic fatigue [2]. One group of plants that is increasingly being researched is various species of *Sesbania*. The *Sesbania* genus has been selected due to its rich phytochemical diversity and its long history of use in traditional medicine. It is widely used in the ayurvedic system of medicine and used in traditional medicine in India for treating a wide range of ailments, such as disorders of the liver, gout, rheumatism, leprosy, and tumors [3]. All parts of this distinctive plant are useful and have a variety of therapeutic uses, where *Sesbania* species are known for their bioactive compounds, such as flavonoids, alkaloids, and saponins, which exhibit a wide range of pharmacological activities, including anti-inflammatory, antioxidant, anticancer, and antimicrobial properties [4]. The broad native range, coupled with its ease of rapid propagation, underscores the economic significance of *Sesbania*. Its diverse applications including use in green manure to enhance soil fertility [5], as a source of animal fodder [6], in traditional medicine for treating various ailments like cellular tissue, circulatory, immune, and sensory system disorders [7], and in bioremediation efforts to restore polluted environments [8], further emphasize its importance and environmental adaptability.

These plants thrive in various conditions, which may contribute to their unique phytochemical profiles that distinguish them from plants in other genera. Given the growing interest in plant-based therapeutics, highlighting the medicinal potential of *Sesbania* species is particularly important for drug discovery and the development of novel therapies. While *Sesbania* species have been traditionally used in various treatments, their application in serious diseases like cancer would require extensive standardization and scientific validation before they could be considered viable therapeutic options in modern medicine.

There are 55 accepted names of species belonging to the genus *Sesbania* (Royal Botanic Gardens, Kew and Missouri Botanical Garden) [9], which is mostly found in Africa and to a lesser extent in Australia, Hawaii, and Asia, and is a member of the Fabaceae family [10].

*Sesbania grandiflora*, *Sesbania cannabina* (*aculeata*), and *Sesbania sesban* (*aegyptiaca*) are some of the most researched species of the *Sesbania* genus. *S. grandiflora*, also known as *Agati grandiflora* L., vegetable-humming bird, and West Indian pea [11], is native to India and Indonesia and is a perennial, evergreen or deciduous, legume tree that can grow to a height of up to 10–15 m; its roots are extensively nodulated, and in wet conditions it can develop floating roots. The pinnately compound leaves are characterized by the presence of 20 to 50 oblong leaflets measuring 1–4 cm in length and 0.5–1.5 cm in width. The maximum length of the leaves is 30 cm. The flowers are arranged in axillary racemes and may be white, pink, crimson, or yellowish in color. The pods, which are glabrous and indehiscent, measure between 50 and 60 cm in length and hang vertically. Additionally, the fruit contains 15–50 dark brown seeds, measuring 5 mm in length and 2.5–3 mm in width [12]. *S. grandiflora* has been used in folk medicine for the treatment of dysentery, stomatitis, fever, smallpox, sore throat, and headache as well as tuberculosis, anemia, and microbial infections [13]. In traditional medicine, *S. grandiflora* leaves are used as a diuretic, purgative, anthelmintic, and hepatoprotective agent [14].

*S. cannabina*, also known as *S. aculeata* Poir., sesbania pea, and corkwood tree [11], has compound, alternating leaves with up to 35 leaflet pairs; and pear-shaped, yellow flowers with a calyx that is 3–5.5 mm long, a standard that is 6–10 mm tall, and wings that are yellow but not streaked with purple. Its pod is 12–20 cm long and 2–3 mm wide, with a pale to yellowish brown color [15]. In traditional medicine, the seeds of *S. cannabina* and flour are combined to treat wounds, ringworm, and other skin conditions. When prepared as a tea, the dried leaves have been shown to possess anti-tumor, antimicrobial, anti-helminthic, and contraceptive properties [16]. *S. cannabina* leaves are employed in the treatment of a plethora of ailments, including stomatitis, fever, headaches, diarrhea, eye infections, oral lesions, and sore throats. They can be chewed for the purpose of cleansing the mouth and throat. Additionally, they possess properties that facilitate bowel movements, increase urine production, induce vomiting, stimulate menstruation, promote laxation, enhance general health, and reduce fever [17].

Sesban (*S. sesban* (L.) Merr.), also known as *S. aegyptiaca* Poir., *S. confaloniana* Chiov., and Egyptian Pea, is native to Egypt [18] and is a perennial legume tree with the potential to reach a height of up to 8 m. It has a rapid growth rate. The plant is distinguished by a shallow root system and stems that can reach up to 12 cm in diameter. The pinnately compound leaves are composed of six to twenty-seven leaflets, with each pair of leaflets being distinct. The leaflets are linear-oblong in shape and measure 26 mm in length by 5 mm in width. The inflorescences are composed of racemes measuring 30 cm in length, which contain two to twenty flowers, exhibiting a yellow hue with streaks of brown or purple. The fruits are characterized by straight or slightly curved pods, which hold between 10 and 50 seeds. The pods reach a length of approximately 30 cm [19]. Traditional medicine utilizes the plant’s seed, bark, and leaves. Seeds are used for skin conditions, substantial menstrual flow, spleen enlargement, and diarrhea. The leaves are used as an antimicrobial agent, an anthelmintic, and to treat inflammatory rheumatic edema [20]. The plant has also shown activity as an antidiabetic, spermicidal, and sleeping aid agent [21]. The application of a poultice prepared from the leaves of *S. sesban* has been demonstrated to facilitate the healing of inflammatory rheumatic swellings, boils, and abscesses [22]. This review emphasizes the anticancer potential of *Sesbania* species, focusing on their phytochemical composition, and their bioactive compounds’ mechanisms of action as anticancer agents, such as apoptosis induction and cell cycle regulation.

## 2. Methodology

A systematic literature review was conducted to investigate the anticancer potential of *Sesbania* species, focusing on their bioactive compounds and their mechanisms of action. To ensure comprehensive coverage, multiple scientific databases were queried, including PubMed, Scopus, Web of Science, and Google Scholar. The search process utilized a combination of specific keywords such as “*Sesbania* anticancer”, “flavonoids *Sesbania*”, “caspase pathway *Sesbania*”, and “bioactive compounds anticancer *Sesbania*”. These keywords were chosen to capture studies related to the anticancer properties, bioactive compounds, and cellular mechanisms of *Sesbania* species. The selection criteria were defined to ensure the inclusion of relevant studies while minimizing bias. Only peer-reviewed articles published in English were considered. Studies that focused on experimental and clinical evidence of anticancer activity related to *Sesbania* were included. The initial search results underwent a systematic screening process conducted independently by all the authors. This review approach ensured that the selection was unbiased and that duplicate studies were removed. Following the screening, studies meeting the inclusion criteria were analyzed in detail and discussed in the context of their findings related to *Sesbania* species and their anticancer effects. This transparent approach allowed for a focused and comprehensive synthesis of the literature.

## 3. *Sesbania* Genus: Selected Species and Their Distribution

### 3.1. Sesbania Adans

*Sesbania* Adans is an accepted genus belonging to Fabaceae. The native range of this genus is tropical and subtropical biomes [23]. Its vernacular name is Sesaban. The plants’ habit is a shrub or tree. According to Legumes of the World Online (2005), the species is known to flourish in a multitude of habitats, including riverine forests, woodlands, wooded grasslands, lake margins, riverbanks, and coastal areas [24,25]. Additionally, it is found in seasonally wet, flooded, or swampy habitats. The plant is utilized for a variety of purposes, including forage, fiber (from the bark), wood, paper, cover crops, green manure, medicinal, and ornamentals. Some species meet these purposes, including *S. grandiflora*, *S. bispinosa* (Jacq.) W.Wight, and *S. exaltata* (Raf.) Cory. Conversely, other species are invasive weeds and are poisonous to livestock, such as *S. punicea* Benth.

### 3.2. Sesbania sesban (L.) Merr.

**Figure 1:**
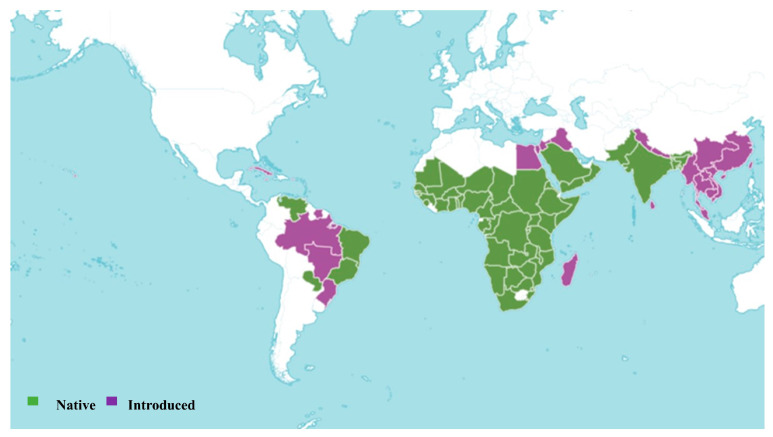
Global distribution of *Sesbania sesban* (L.) Merr. according to Plants of the World Online [24].

*Sesbania sesban* is an accepted species, first published in Philipp. J. Sci., C 7: 234 (1912). The native range of this species is tropical and south Africa, the Arabian Peninsula, and the Indian Subcontinent (Figure 1). It has been introduced to many other regions in South America, Madagascar, Sinai, West Himalaya, and others, as shown in Figure 1. It is a shrub or tree and grows primarily in the seasonally dry tropical biome [24,25]. The Egyptian Sesban *Sesbania sesban* was most recently assessed for the IUCN Red List of Threatened Species in 2019, and is listed as Least Concern and the population is assumed to be stable. The plant is a widespread species with no known major threats. However, the species needs to be monitored as the level of harvesting is increasing [26]. It is used as fiber, food, forage, medicine, and wood [24]. Its cytotoxic effects highlight its pharmaceutical relevance and underscore the importance of continued investigation into its bioactive properties.

### 3.3. Sesbania grandiflora (L.) Poir.

**Figure 2:**
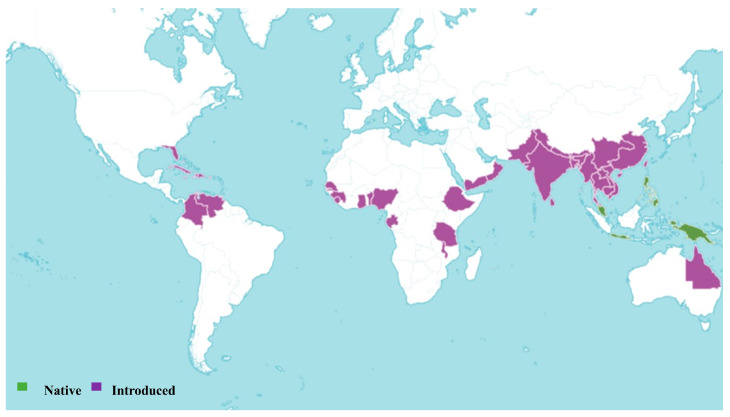
Global distribution of *Sesbania grandiflora* (L.) Poir. according to Plants of the World Online [24].

*Sesbania grandiflora* is an accepted species, first published in J.B.A.M.de Lamarck, Encycl. 7: 127 (1806). The native range of this species is Malesia to New Guinea. It is cultivated and naturalized in Colombia (Figure 2). The plant is a shrub or tree and grows primarily in the wet tropical biome [24]. *S. grandiflora* was most recently assessed for the IUCN Red List of Threatened Species in 2023, and is listed as Data Deficient as the native range of the taxon cannot be established [27,28]. This species has environmental and social usage, and is commonly cultivated for medicinal uses, animal food, ornamental purposes, and fuel [28]. Despite these uncertainties, its broad phytochemical composition and its applications in traditional medicine underscore its potential for pharmaceutical research.

### 3.4. Sesbania cannabina (Retz.) Poir.

**Figure 3:**
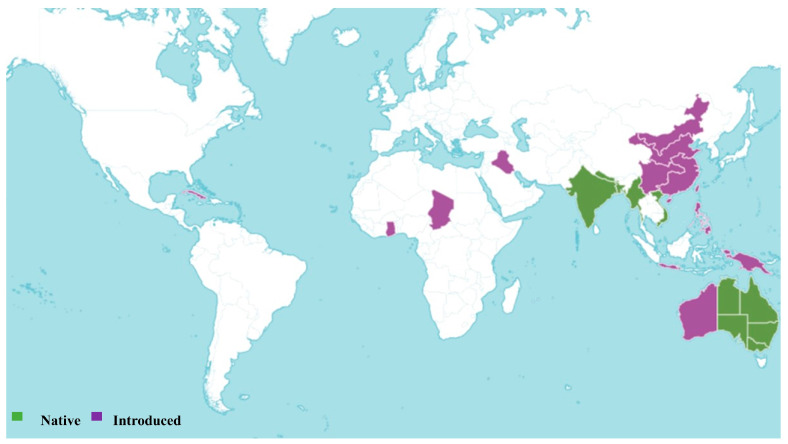
Global distribution of *Sesbania cannabina* (Retz.) Poir. according to Plants of the World Online [24].

*Sesbania cannabina* is an accepted species, first published in J.B.A.M. de Lamarck’s Encyclopédie (7: 130) in 1806. This species is native to the Indian Subcontinent, Indo-China, and Australia (Figure 3), and typically grows as an annual, perennial, or subshrub in seasonally dry tropical biomes [23,25]. *S. cannabina* has been assessed for the IUCN Red List of Threatened Species and is currently listed as Least Concern, although the information requires updating [26,28]. The plant is utilized for a variety of purposes, including the production of chemical products, environmental applications, fiber, and forage [19,25]. Homotypic synonyms for this species include *Aeschynomene cannabina* Retz., *Coronilla cannabina* (Retz.) Willd., and *Sesbania aculeata* var. *cannabina* (Retz.) Baker. The diverse applications and wide native range of *S. cannabina* highlight its ecological and economic significance.

The conservation status of selected species of the *Sesbania* Adans genus, as assessed by the IUCN Red List of Threatened Species, reveals varying levels of concern (Table 1).

## 4. *Sesbania* Adans Propagation

### 4.1. S. sesban

*S. sesban* is usually propagated by seeds. The plant produces 40 seeds within each pod. The seeds are easy to collect and store. Best growth is achieved at 500–2000 mm annual rainfall. Cuttings can also be used for Sesban propagation [29]. As the seed has an impermeable hard coat, scarification is recommended. Scarification of *S. sesban* seeds is known to enhance germination; it is achieved by dipping the seeds in water heated to just below boiling for 30 s. Soaking in warm or cold water for 24 h may also be effective. Plants cultivated for fodder production should be spaced 30–50 cm apart and arranged in rows separated by one meter. It may be necessary to use rhizobia inoculants upon planting. *S. sesban* is known for its outstanding ability to withstand floods, salt, waterlogging, and alkaline environments [30]. Intensive cutting management is not recommended as it shortens the life period of the plant to be 3–5 years [30].

### 4.2. S. grandiflora

*S. grandiflora* is easily and rapidly established, either by sowing seeds or by vegetative propagation using cut stems and branches [31]. The pods are long and narrow; each contains about 15–40 seeds. Seed storage is orthodox, as seeds can survive prolonged periods of freezing and drying while they are being preserved ex situ. The seeds are not hard and usually germinate well without scarification. Pretreatment could include either nicking or scratching the round end of each seed, avoiding contact with the cotyledon [30]. It should be mentioned that 85–90% germination can be achieved when soaking seeds in cold or warm water for 24 h. The seeds can be either sown directly into the soil at the beginning of the rainy season or can be grown first in plastic bags, then after a month might be planted in a field. They should be cultivated in 20–25 cm holes in patches or lines separated by about 1.2 m [30]. Irrigation and weeding are known to promote early growth. *S. grandiflora* is well adapted to humid, hot environments with annual average temperatures of 22–30 °C. It is frost sensitive and cannot tolerate temperatures below 10 °C [31]. The plant thrives in areas that flood periodically and has an exceptional capacity to withstand waterlogging. It also has the ability to tolerate extended dry seasons of up to nine months [30].

### 4.3. S. cannabina

*S. cannabina* is also propagated easily by seeds. Freshly harvested *S. cannabina* seeds possess physical dormancy [32]. A boiling-water scarification treatment for five minutes’ duration is the optimum treatment to overcome this dormancy. Seed germination decreases linearly with the increase in moisture stress. *S. cannabina* prefers consistently moist soil but not waterlogged environments. Checking soil humidity before watering is essential. The plant should be watered thoroughly, ensuring the water reaches the roots. During the growth season, watering once every seven to ten days is the recommended frequency. Reducing watering to once every two to three weeks during the dormant season is preferable to prevent root rot. Adjustments to the water regime are mainly based on the size of the plant and the environmental conditions. Maximum germination typically happens in two conditions: one, with consistent temperatures of 32 or 35 °C; and the other with alternating temperatures of 30/20 and 35/25 °C [32]. With pH 9.0 promoting maximum germination, it germinates well over a wide range of pH values. The highest germination rate is at 1.0 cm burial depth. Deep tillage usually stops *S. cannabina*’s emergence [32].

## 5. *Sesbania* Phytochemistry

Chemicals known as phytochemicals are created by plants throughout their primary and secondary metabolism. They are essential to the growth, development, and defense systems of plants. These compounds are classified into primary and secondary metabolites. Primary metabolites, such as sugars, amino acids, and lipids, are directly involved in the growth and metabolic functions of the plant. In contrast, secondary metabolites are not directly involved in growth but serve important ecological functions, including protection against predators, pathogens, and environmental stressors. Terpenoids, alkaloids, flavonoids, tannins, saponins, and coumarins are important groups of secondary metabolites. They have a wide range of biological functions and are of great interest for their potential therapeutic use in human medicine [33,34].

*S. grandiflora*, and *S. sesbane* have been shown to be rich in secondary metabolites, while *S. cannabina* studies have focused mainly on primary metabolites.

### 5.1. Sesbania grandiflora

*S. grandiflora*, commonly known as the agati or hummingbird tree, exhibits a diverse range of secondary metabolites with potential medicinal and nutritional benefits. The plant produces a variety of compounds, including alkaloids, flavonoids, tannins, saponins, terpenoids, and phenolics, with peak lipid and alkaloid content observed during the summer [35]. The leaves are particularly rich in these compounds compared to the bark and wood [36]. Bio-guided fractionation and HPLC purification have identified specific terpenoids and flavonoids in the leaves, such as vomifoliol, loliolide, quercetin, and kaempferol [37]. The bark contains unique 2-arylbenzofurans (sesbagrandiforian A and B), which are under investigation for their pharmacological effects [38].

*S. grandiflora* exhibits a diverse range of phytochemicals across its different parts, with flavonoids, tannins, alkaloids, saponins, and steroids being particularly prevalent [39]. Flavonoids, characterized using NMR spectroscopy and mass spectrometry (MS), are notably abundant in the leaves, flowers, roots, and seeds, especially in the leaves across various extracts [40,41,42]. Tannins are similarly distributed, being present in the leaves, flowers, and bark [41,43]. Alkaloids are widely detected in the leaves and flowers, with significant concentrations also in the roots. Saponins are found in the leaves and seeds [9,36], whereas steroids are prevalent in the leaves, flowers, and roots [12,14]. This rich phytochemical diversity underscores the potential medicinal applications of *Sesbania grandiflora*, warranting further research into its therapeutic properties [38,44].

Analyzing the phytochemical composition of *Sesbania grandiflora* using HPLC analysis reveals a diverse array of compounds across its different parts. Each part of the plant—bark, root, seed, leaves, and flower—contains a unique combination of phytochemicals. Hydroalcoholic leaf extracts are particularly effective due to their polarity, containing alkaloids, amino acids/proteins, carbohydrates, glycosides, phenols, saponins, steroids, sugars, tannins, terpenoids, and flavonoids such as quercetin and kaempferol. These two flavonoids are known for their potent antioxidant and anti-inflammatory properties. Quercetin, for instance, has been extensively studied for its ability to scavenge free radicals and inhibit oxidative stress, while kaempferol exhibits both antioxidant activity and anticancer potential by modulating signaling pathways such as PI3K/AKT and NF-κB. Additionally, the leaf extracts are particularly rich in polyphenols, as quantified using the Folin–Ciocalteu method, further underlining their antioxidant potential and their potential role in mitigating oxidative damage in biological systems [36,45]. Carbohydrates are also prominently found in the leaves and flowers [14,35,36]. The roots contain distinctive compounds like isovestitol, medicarpin, sativan, and belulinic acid, while the seeds are distinguished by tocopherols (α, β, γ, δ) and phytosterols (β-sitosterol), emphasizing their antioxidant properties [46]. Additionally, vitamin C is present in both the leaves and flowers, enhancing their nutritional value [45].

**Figure 4:**
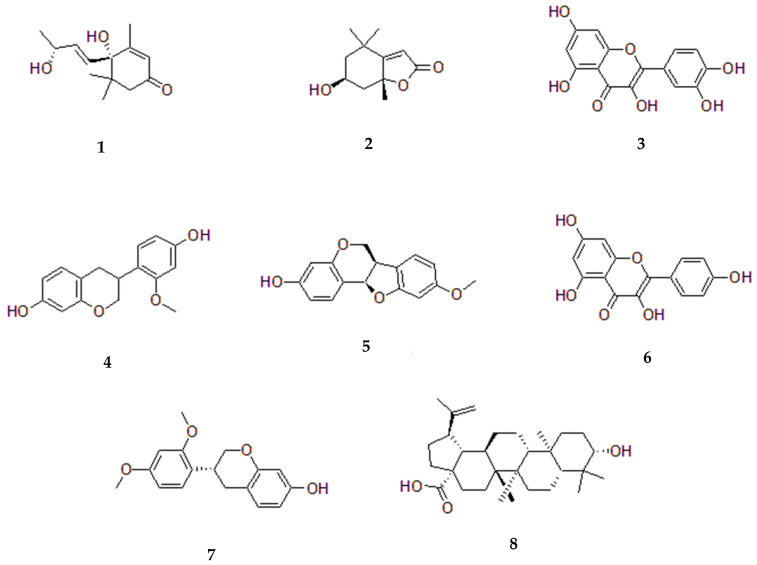
Structures of selected compounds isolated from *Sesbania grandiflora*: Vomifoliol (**1**), Loliolide (**2**), Quercetin (**3**), Isovestitol (**4**), Medicarpin (**5**), Kaempferol (**6**), Sativan (**7**), and Belulinic (**8**).

This analysis indicates that while common phytochemicals are widely distributed throughout *S. grandiflora*, certain plant parts are particularly rich in unique and significant compounds, enhancing their potential utility in various therapeutic and nutritional applications. Table 2 details the phytochemical compounds identified in *S. grandiflora*, the specific plant parts used for their extraction, and the solvents employed in the extraction process, and Figure 4 shows the structure of some of these compounds.

### 5.2. Sesbania sesban

The phytochemical composition of *S. sesban* varies significantly across its different plant parts and the extraction solvents used, as detailed in the provided data. Starting with the bark, various extracts including petroleum ether, chloroform, ethanol ether (diethyl ether), and aqueous extracts reveal common phytochemicals such as carbohydrates, alkaloids, phytosterols, saponins, glycosides, and phenolic compounds. Notably, aqueous extracts uniquely contain sugars like glucose, fructose, erythritol, arabinitol, and myo-inositol, suggesting a distinct chemical profile accessible through this solvent [49,50].

Moving to the root, ethyl acetate and n-butanol-saturated extracts yield oleanolic acid 3-β-D-glucuronide as a significant phytochemical component [20]. Sterols, triterpenes, and flavonoids abound in *S. sesban* wood petroleum ether and chloroform ethyl acetate extracts, adding to the plant’s chemical variety [51].

The leaves show a wide range of phytochemicals depending on the extraction technique. Extracts from 60 to 80 °C using methanol, chloroform, and petroleum ether show the presence of proteins, gums, anthraquinone glycosides, sterols, saponins, flavonoids, alkaloids, fats and oils, and other substances [52]. Triterpenoids, sugars, proteins, amino acids, vitamins, glycosides, tannins, and saponins, detected using HPLC, are all highlighted in the leaves’ aqueous extracts [53]. Furthermore, specific components like campesterol, cholesterol, beta-sitosterol, and distinct proteins and tannins are uniquely identified in aqueous extracts as the most abundant metabolites, emphasizing the variability in chemical profiles based on the extraction solvent [53,54].

Extracts of flowers and blossoms of *S. sesban*, identified using GC-MS (gas chromatography–mass spectrometry), show unique phytochemical profiles as well. Methanol and acidified methanol extracts from flowers contain anthocyanins, phenols, and flavonoids, whereas blossoms yield cyanidin and delphinidin glucosides, specifically in aqueous extracts, which were identified using reversed-phase high-performance liquid chromatography analysis [20,21,49,55]. Dust (pollen) and dust tubes are noted for containing alpha-ketoglutaric, oxaloacetic, and pyruvic acids in their aqueous extracts, highlighting specialized chemical constituents associated with reproductive parts [21,55].

Lastly, lignin extracted from various parts of the plant reveals guaiacyl, syringyl, p-hydroxyphenylpropane, and kaempferol in aqueous extracts, contributing to the overall chemical diversity observed in *S. sesban* [50]. The phytochemicals found in *Sesbania* species, such as flavonoids, alkaloids, and saponins, possess unique structural features that contribute to their biological activities [56]. For instance, flavonoids in *Sesbania* exhibit strong antioxidant properties, which play a significant role in scavenging free radicals and reducing oxidative stress, a key factor in cancer progression. The structural diversity of these compounds, particularly the presence of hydroxyl groups and conjugated double bonds, enhances their ability to interact with multiple molecular targets, including DNA and proteins involved in cell signaling pathways.

In addition, the alkaloids found in *Sesbania* have demonstrated the ability to interfere with cellular processes such as DNA replication and mitosis, making them particularly effective in inhibiting cancer cell proliferation. The saponins, on the other hand, exhibit membrane-disruptive properties, which may contribute to the induction of apoptosis in cancer cells.

**Figure 5:**
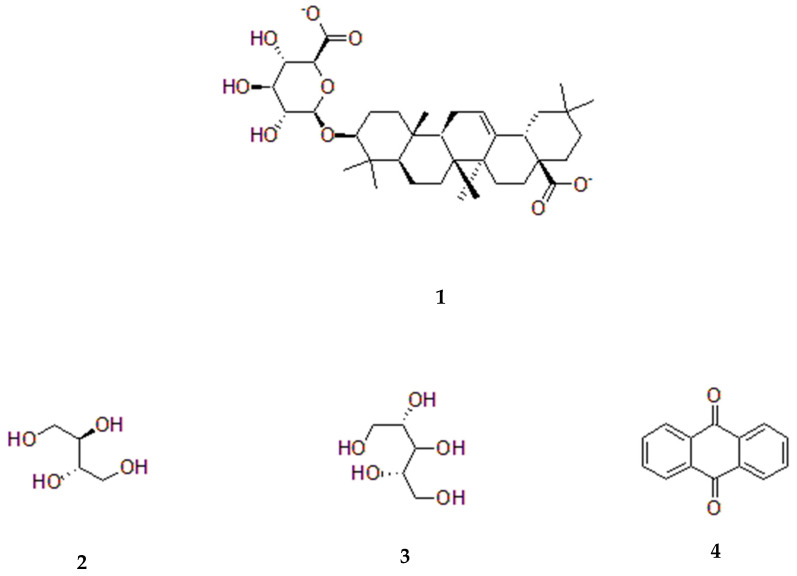
Structures of selected compounds isolated from *Sesbania sesban*: Oleanoic acid 3-O-glucuronide (**1**), Erythritol (**2**), Arabinitol (**3**), and Anthraquinone (**4**).

In summary, *S. sesban* demonstrates a rich and varied phytochemical profile across its different plant parts, influenced by the extraction solvent employed, as shown in Table 3. The chemical structures of some of these important compounds are displayed in Figure 5. For example, oleanolic acid showed antiproliferative effect against the K562 chronic myeloid leukemia (CML) cell line [57]. Erythritol could be of significant advantage as a preferred sugar substitute for diabetic or pre-diabetic people for reducing their risk of diabetic complications [58]. In addition, anthraquinones and their derivatives are produced as secondary metabolites in plants and many other organisms. They are potent aromatic compounds known for their remarkable biological activities such as anticancer, antifungal, antibacterial, diuretic, anti-inflammatory, and relieving constipation [59,60]. These findings underscore the potential pharmacological and nutritional significance of this plant species, prompting further investigation into its bioactive compounds and their diverse applications in medicine and agriculture.

## 6. Cytotoxic Effect of *Sesbania* Species

### 6.1. Cytotoxicity of Sesbania grandiflora

**Figure 6:**
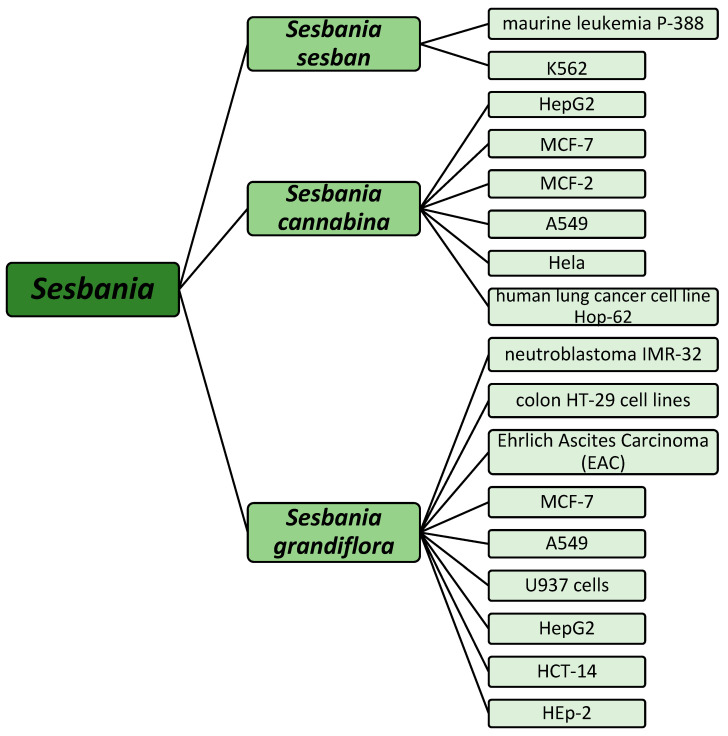
Overview of cell lines treated by different *Sesbania* species.

Laladhas et al. (2010) [60] extracted a protein fraction from the flower of the medicinal plant *S. grandiflora*, known as SF2, which has shown effectiveness against cancer cell lines through mechanism-based research [61] Figure 6. The cytotoxic effect of SF2 was investigated in two tumor cell lines from mice with ascites and in human cancer cell lines from different sources. In Dalton’s lymphoma ascites (DLA) and colon cancer cells (SW-480), DNA fragmentation and the externalization of phosphatidylserine were markers indicating that SF2 reduced cell growth and induced cell death. The activation of caspases 3, 8, and 9, cleavage of poly(ADP-ribose) polymerase (PARP), and release of cytochrome c are indicative of apoptosis-induced cell death. Specifically, SF2 appears to engage both the extrinsic and intrinsic apoptotic pathways. The activation of caspase-8 suggests the involvement of the extrinsic pathway, likely triggered by death receptor signaling, which subsequently activates downstream effector caspases like caspase-3. On the other hand, the release of cytochrome c from mitochondria and the activation of caspase-9 highlights the role of the intrinsic mitochondrial pathway. Cytochrome c release, facilitated by mitochondrial membrane permeabilization, forms part of the apoptosome complex with Apaf-1, which activates caspase-9. Caspase-3, as the executioner caspase, then cleaves substrates such as PARP, leading to DNA fragmentation and cell death. Together, these mechanisms underline the dual apoptotic pathways triggered by SF2, contributing to its potent cytotoxic effects in tumor cells. Caspases play critical roles in the execution phase of apoptosis by cleaving essential cellular components. Additionally, compounds derived from *Sesbania* induce the generation of reactive oxygen species (ROS), leading to mitochondrial dysfunction. This dysfunction results in the release of cytochrome c into the cytosol, further activating the caspase cascade [4].

In response to phorbol myristate acetate (PMA) induction, SF2 mechanistically downregulated the transcription factor NF-κB, which controls the expression of genes encoding proteins essential for cell regulation and growth control. To further explain its anticancer activity, SF2 also blocked anti-apoptotic proteins such as cyclooxygenase-2, p-Akt, and Bcl-2, which were activated by the tumor promoter PMA. Ex vivo studies using solid tumor models and ascites provided strong support for the in vitro findings, as SF2 therapy decreased tumor volume and increased survival in tumor-bearing mice. Through in vivo toxicological testing, the pharmacological safety of SF2 was established, suggesting its potential as an anticancer treatment candidate [4] Figure 6.

Sreelatha et al. (2011) [4] administered ethanol extracts of *Sesbania grandiflora* (EESG) to Swiss albino mice at doses of 100 and 200 mg/kg body weight intraperitoneally to test their anticancer effects on the Ehrlich ascites carcinoma (EAC) cell line [62]. The extracts increased the lifespan of EAC-bearing mice and significantly decreased tumor volume, viable cell count, and tumor weight (*p* < 0.01). In mice treated with EESG, hematological parameters such as RBC count, hemoglobin level, and lymphocyte count returned to normal. The levels of lipid peroxidation were significantly reduced (*p* < 0.05) by the extracts, while the levels of GSH, SOD, and CAT were significantly elevated (*p* < 0.05) [4].

Roy et al. (2013) [63] assessed the cytotoxic potential of fraction F2 of *Sesbania grandiflora* using an MTT-based cell viability experiment. On U937 cells, it worked best with an *IC*_50_ of 18.6 μg/mL. An increase in annexin V positivity inhibited growth. Flow cytometry showed that this was accompanied by a decrease in oxygen consumption and an increase in the generation of reactive oxygen species, both of which were corrected by the antioxidant NAC. Flow cytometry and cytochrome c release measurements indicated that pro-apoptotic protein expression was stimulated while anti-apoptotic protein expression was inhibited, resulting in mitochondrial depolarization. It is intriguing that despite these molecular features associated with apoptosis, F2 was still able to alter Atg protein levels and initiate LC3 processing. The presence of autophagy was demonstrated by electron microscopy, which also showed that this was accompanied by the formation of autophagic vacuoles. Finally, translocation of AIF to the nuclei and programmed cell death is induced by activation of the caspase cascade by F2. Hoechst 33,258 staining showed that this leads to degradation of the DNA repair enzyme poly (ADP-ribose) polymerase, resulting in DNA damage and cell death. F2 demonstrated selective cytotoxicity towards U937 cancer cells without affecting normal, benign cells. Specifically, F2 was unable to inhibit the proliferation of human peripheral blood mononuclear cells (PBMCs), even at a concentration as high as 100 µg/mL, indicating that it does not confer toxicity to healthy normal cells. In contrast, F2 caused marked growth inhibition and a decrease in cell viability in U937 cancer cells in a dose- and time-dependent manner. This selective cytotoxicity suggests that F2 may have potential as a therapeutic agent, targeting cancer cells while sparing normal cells [63].

Ponnanikajamideen et al. [62] compared *Sesbania grandiflora* leaf extracts to common commercial anticancer drugs and reported on their potential cytotoxic activity. The chemical composition of the leaf extracts included alkaloids, flavonoids, glycosides, tannins, anthraquinones, steroids, and terpenoids. The commercial anticancer drugs used for comparison were doxorubicin and cisplatin. *S. grandiflora* leaf extracts in water, ethanol, and acetone showed in vitro anticancer efficacy against several human cancer cell lines, including neuroblastoma (IMR-32) and colon (HT-29). The MTT technique was used to evaluate the potential cytotoxic properties of *S. grandiflora* leaf extract. The extract concentration ranged from 50 to 300 μg/mL during the activity. According to the investigation, all the extracts had an *IC*_50_ of 200 μg/mL against the neuroblastoma (IMR-32) and colon (HT-29) cell lines. When extract concentration was increased, cell viability decreased. *S. grandiflora* extracts reduced cell viability and induced apoptosis in the neuroblastoma (IMR-32) and colon (HT-29) cell lines in a dose-dependent manner. These in vitro results point to *S. grandiflora* having considerable therapeutic effects on human neuroblastoma (IMR-32) and colon (HT-29) cell lines [62].

Pajaniradje et al. [64] examined five distinct solvent fractions from *S. grandiflora* leaves in their study on cancer cell lines including MCF-7, HepG2, Hep-2, HCT-15, and A549. It was discovered that *S. grandiflora*’s methanolic fraction had strong antiproliferative effects, particularly on the human lung cancer cell line A549. A549 cells treated with methanolic fraction had activated caspase-3, which caused apoptosis or cell death. The DNA laddering, DAPI staining, and decline in mitochondrial membrane potential all contributed additional confirmation to the apoptotic mode of cell death. The large quantities of ROS intermediates found by DCF-DA labeling may have contributed to the triggering of apoptosis. On treatment with the methanolic fraction, A549 cells showed decreased levels of cyclin D1 and decreased NFkB activation, providing a clue as to the potential mode of action. These findings demonstrate the potential of S. grandiflora as a source of intriguing candidate molecules for the treatment of cancer, particularly lung cancer [64].

Padmalochana and Rajan (2015) discussed the possible anticancer properties of *Sesbania grandiflora* leaf extracts and contrasted them with common commercial anticancer medications. *S. grandiflora* leaf extracts in water, ethanol, and acetone demonstrated in vitro anticancer activity against many human cancer cell lines, including HEp2 (human larynx carcinoma cell line). The MTT technique was used to evaluate the leaf extract from *S. grandiflora*’s potential anticancer properties. The extract concentration used for the activity ranged from 50 to 300 μg/mL. According to the research, all the extracts had an *IC*_50_ of 200 μg/mL against HEp2 (human larynx carcinoma cell line) cell lines. At 200 μg/mL, the treatment with water, ethanol, and acetone extracts reduced cell viability by up to 50%. The extract significantly and dose-dependently decreased the viability of cells. When compared to other extracts, the treatment of HEp2 (human larynx carcinoma cell line) cell lines with ethanol extract dramatically reduced the viability of cells at 200 μg/mL. The extract concentrations of 50 μg/mL, 100 μg/mL, 150 μg/mL, 200 μg/mL, 250 μg/mL, and 300 μg/mL that were applied to the cells caused a reduction in the number of viable cells as well. The ethanol extract had the greatest cytotoxic effect. The presence of many phytoconstituents, including flavonoids, alkaloids, and steroids, causes this variance in action. Flavonoids, alkaloids, phenols, polyphenols, and other derivatives have been linked to anticancer activity, they are phytochemicals that actively prevent cells from synthesizing proteins either by destroying DNA or by inhibiting it at the global level, which may affect cell viability. Alkaloids and flavonoids, found in high concentrations in the ethanol extract, play a key role in the death of cancer cells. The drugs cause apoptosis and necrosis, which lead to cell death [65] Figure 6 and Table 4.

### 6.2. Cytotoxicity of Sesbania cannabina

Zhou et al. (2018) [66] extracted mannanase hydrolysate, a native galactomannan (GalM), and some of its hydrolysates (GalM40, GalM50 and GalM65) from *S. cannabina* seeds using water extraction and ethanol precipitation. With a molecular weight of 1.42 × 10^6^ Da, GalM was determined to be a 1,4-linked d-mannose polymer with a single 1,6-linked d-galactose side chain in a 2.4:1 M ratio using HPAEC, NMR, FT-IR, and HPGPC. The MTT assay revealed that these four fractions significantly inhibited A549, Hela, HepG2, and MCF-7 in a dose-dependent manner. Of those fractions, GalM40 had the highest activity and the second-highest MW (1.47 × 10^4^ Da). An immune-histochemical investigation has suggested that its significant inhibitory effect on the growth of human cancer cells may be due to its ability to upregulate the expression of caspase-12 [66].

When compared to standard chemotherapeutic agents, *Sesbania cannabina* derivatives exhibit several distinct advantages and differences. First, standard chemotherapeutics, such as doxorubicin or cisplatin, target cancer cells through mechanisms like DNA damage, inhibition of mitosis, or induction of oxidative stress, often leading to broad cytotoxicity in both cancerous and healthy cells. In contrast, GalM derivatives appear to have more selective mechanisms, such as modulation of apoptosis through caspase pathways, potentially resulting in reduced off-target effects [63]. Additionally, standard agents frequently induce multidrug resistance (MDR) due to efflux pumps and other adaptive cellular responses, whereas natural products like GalM40 may bypass these resistance mechanisms by targeting alternative pathways, such as ER stress. However, the lower molecular weight and polysaccharide nature of GalM40 suggest a distinct pharmacokinetic profile that may limit its systemic bioavailability compared to small-molecule chemotherapeutics.

Using the SRB test, Mehta et al. (2019) [67] assessed the cytotoxic potential of extracts from *S. aculeata* against the human lung cancer cell line Hop-62. *S. aculeata* extracts demonstrated good in vitro antioxidant activity when tested using several methods; the highest activity was found in the seed coat extract, which ranged from 71 to 67%. Similar to this, only the extracts from the seeds’ coats showed any cytotoxic efficacy against the tested cancer cell line. According to the findings, methanolic extracts may be a source of potential antioxidants and cytotoxic compounds. At a concentration of 80 μg/mL, the seed coat extract was found to have cytotoxic potential against the cell line tested, inhibiting cell growth with an average viability of 70%, while other extracts showed little to no effect. The disruption of cell homeostasis by biochemical and genetic changes can be measured using a variety of techniques, including enzyme release, cell viability, survival, and death assays. The SRB assay yields results that vary depending on the number of cells or the rate of protein turnover of the cells examined. The most noticeable outcome of toxicant exposure in cells is a change in morphology. The findings demonstrate that as the extract’s concentration is increased, the cytotoxicity of the seed coat extract also increases [67].

Fu et al. (2021) [68] used *S. cannabina* (Retz.) Poir stems to isolate two novel 2-arylbenzofuran compounds, sesbcanfuran A and B (**1** and **2**), as well as six more 2-arylbenzofuran derivatives (**3**–**8**). Detailed spectroscopic techniques were used to reveal the structures of these substances. All the chemicals’ inhibitory effects on three cancer cell lines MCF-2, HeLa, and A549 were assessed. The MCF-2 and A549 cell lines were most susceptible to compound **2** (*IC*_50_ = 1.5 μM and 4.8 μM, respectively), while its activity against HeLa cells was low. All three cell lines were somewhat responsive to compounds **1** and **4**. Against HeLa, MCF-7, and A549 cells, compounds **3** and **5**–**8** displayed moderate inhibitory effects or exhibited no activity. The other compounds were deemed inactive since their *IC*_50_ values were greater than 40 μM. According to those findings, compounds **1**, **2**, and **4** are more potent against HeLa, MCF-7, and A549 cells than compounds **3** and **5**–**8** [68] as summarized in Table 4.

### 6.3. Cytotoxicity of Sesbania sesban

**Figure 7:**
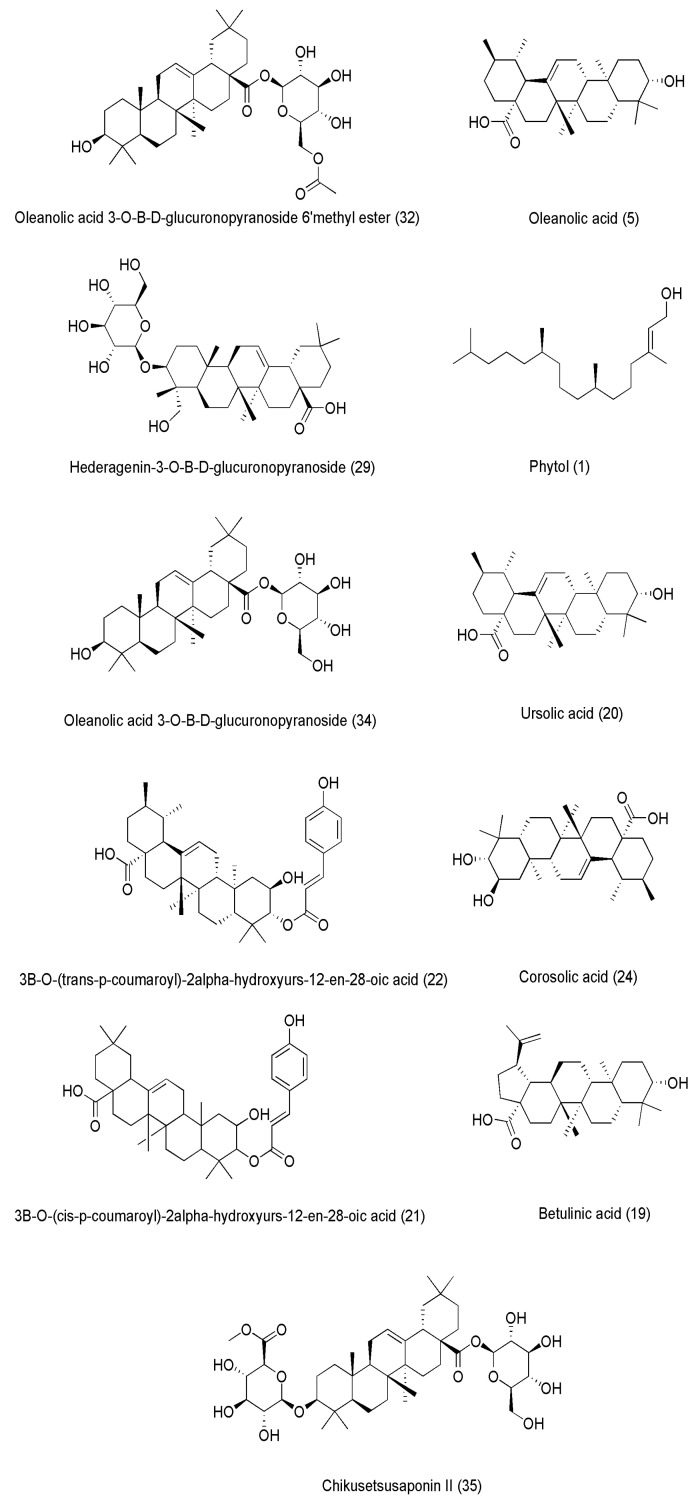
Structures of selected compounds isolated from *S. sesban* (Egyptian river hemp) that showed cytotoxic activity against the K562 cell line in an aqueous ethanolic extract according to Abdelgawad et al. (2022) [69].

*S. sesban* (Egyptian river hemp) showed cytotoxic activity against the K562 cell line in an aqueous ethanolic extract according to Abdelgawad et al. (2022) [69]. Using bioguided fractionation, a new molecule, hederatriol 3-O-D-glucuronic acid methyl ester, and thirty-four previously known compounds were extracted from the hydroethanolic extract of SS leaves. Four chemicals (oleanolic acid 3-*O*-*β*-D-glucuronopyranoside 6′ methyl ester (32), oleanolic acid (5), hederagenin-3-*O*-*β*-D-glucuronopyranoside (29), and phytol (1)) showed milder effects (*IC*_50_ = 56.4, 67.6, 83.3, and 112.3 μM, respectively), whereas seven substances (oleanolic acid 3-*O*-*β*-D-glucuronopyranoside (34), 3*β*-*O*-(*trans*-*p*-coumaroyl)-2*α*-hydroxyurs-12-en-28-oic acid (22), ursolic acid (20), corosolic acid (24), 3*β*-*O*-(*cis*-*p*-coumaroyl)-2*α*-hydroxyurs-12-en-28-oic acid (21), betulinic acid (19), and chikusetsusaponin II (35)) showed strong antiproliferative effects (*IC*_50_ = 22.3, 30.8, 31.3, 33.7, 36.6, 37.5, and 41.5 μM, respectively). The antileukemic effects of these compounds may result from the suppression of the Smad, Wnt, and E2F signaling pathways. The structures of these lead compounds are shown in Figure 7 and Table 4.

At the molecular genetics level, a mechanistic examination was also carried out into several transcription factors and signaling pathways implicated in the development of cancer. According to the findings, chemicals (22) and (21) specifically inhibited the Wnt pathway (*IC*_50_ values: 3.8 and 4.6 μM, respectively), whereas compound (**22**) specifically inhibited the Smad pathway (*IC*_50_ value: 3.8 μM). Smad and E2F pathway signaling was significantly affected by compound (**34**), with an *IC*_50_ value of 5 μM. By docking against various targets connected to the K562 cell line, the bioactive compounds were further examined in silico. The outcomes demonstrated that compounds (**22**) and (**34**), with docking scores of 7.81 and 9.30 kcal/mole, respectively, had a considerable binding affinity towards topoisomerase. EGFR-tyrosine kinase was strongly bound by compounds (**22**) and (**34**) (docking scores of 7.12 and 7.35 kcal/mole, respectively). Additionally, compound (**34**), with a docking score of 7.05 kcal/mole, demonstrated a high affinity for binding to Abl kinase [69].

**Figure 8:**
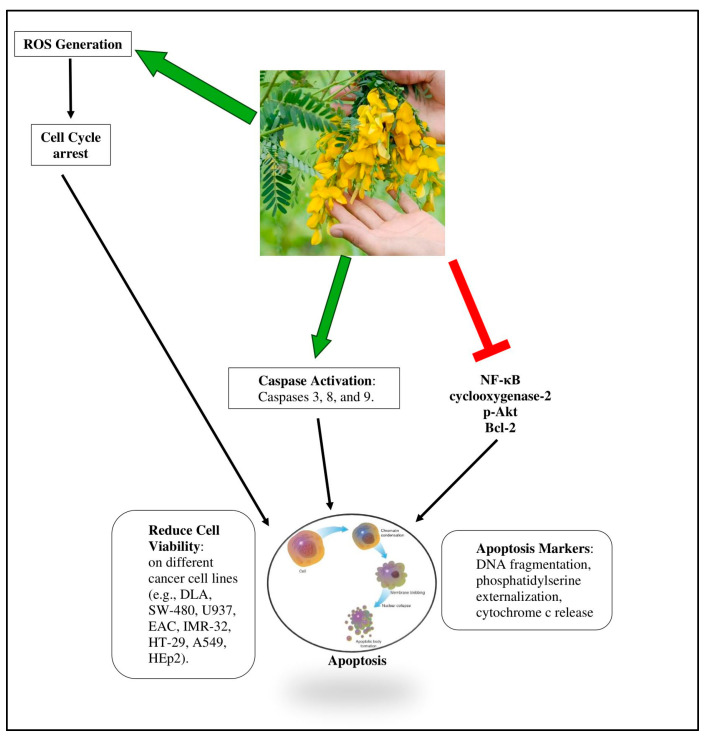
Comprehensive overview of anticancer effects of *Sesbania* species.

Dianhar et al. (2014) [70] used *S. sesban* leaf methanol extract to separate the secondary metabolites and test their cytotoxicity against murine leukemia P-388 cells. The isolated 3-hydroxy-4′,7-dimethoxyflavone, a novel substance from nature, was obtained and identified. This chemical was produced by separating the methanol extract using a variety of chromatographic procedures, including vacuum liquid chromatography and radial chromatography. 1D NMR (1H-NMR and 13C-NMR) and 2D NMR (HMBC) were used to determine the structure of an isolated molecule. By using the MTT assay to test their cytotoxicity against murine leukemia P-388 cells, the methanol extract and compound **1** both demonstrated *IC*_50_ values of 60.04 μg/mL and 5.40 μg/mL, respectively [70]. Figure 8 shows a comprehensive overview of anticancer effects of *Sesbania* species.

**Table 4:** Summary of articles about *Sesbania* species and their anticancer effects.

Plant Species	Part Used	Type of Study	Type of Extract	Results	Reference
* Sesbania grandiflora *	Flower	In vitro: human cancer cell lines and two ascites tumor cell lines from mice	Protein fraction: SF2	Limited the growth of cells and brought about apoptosis by the activation of caspases 3, 8, and 9, cleavage of poly (ADP-ribose) polymerase, and release of cytochrome c, all of which point to the death of cells through apoptosis.	[53]
		In vivo: Swiss Albino mice bearing Ehrlich ascites carcinoma (EAC)	Ethanol extracts at doses of 100 and 200 mg/kg body weight	Considerable reductions in tumor weight, viable cell count, and volume.	[54]
	Leaf	In vitro: neuroblastoma (IMR-32), and colon (HT-29) cell lines	Extract concentration ranged from 50 to 300 μg/mL	All the extracts have an *IC*_50_ of 200 μg/mL against the neuroblastoma (IMR-32) and colon (HT-29) cell lines. With increasing extract concentration cell viability decreased.	[21]
	Leaf	In vitro: MCF-7, HepG2, Hep-2, HCT-15, and A549	Methanolic extract	Extracts activated caspase-3, increased ROS intermediates, as well as decreased the levels of cyclin D1, which caused apoptosis.	[59]
		In vitro: U937 cells	Protein fraction: F2	Extract had an *IC*_50_ of 18.6 μg/mL. Cytotoxicity was achieved by decreasing oxygen consumption and increasing reactive oxygen species formation as well as release of cytochrome c, which activates pro-apoptotic proteins.	[56]
	Leaf	In vitro: HEp2 (human larynx carcinoma cell line)	The concentrations of water, ethanol, and acetone extracts utilized in the activity varied from 50 to 300 μg/mL	All of the extracts have an *IC*_50_ of 200 μg/mL against HEp2 (human larynx carcinoma cell line) cell lines.	[60]
* Sesbania cannabina *	Seed	In vitro: A549, Hela, HepG2, and MCF-7	Water extraction and ethanol precipitation	Owing to their capacity to elevate caspase-12 expression, all fractions dose-dependently suppressed the proliferation of the targeted cells.	[61]
	Seed coat	In vitro: human lung cancer cell line Hop-62	Methanolic extract	Extract prevented cell growth by disrupting cell homeostasis as well as changes in cell morphology.	[67]
	Stem	In vitro: MCF-2, HeLa, and A549	Isolates of two novel 2-arylbenzofuran compounds, sesbcanfuran A and B (**1** and **2**), and six 2-arylbenzofuran derivatives (**3**–**8**)	Compounds **1**, **2**, and **4** are more potent against HeLa, MCF-7, and A549 cells than compounds **3** and **5**–**8**.	[68]
* Sesbania sesban *	Leaf	In vitro: murine leukemia P-388 cells	Methanol extract and isolated 3-hydroxy-4′,7-dimethoxyflavone	Methanol extract and compound **1** both demonstrated *IC*_50_ values of 60.04 μg/mL and 5.40 μg/mL, respectively.	[70]
	Leaves	In vitro: K562 cell line	Aqueous ethanol extract	Cytotoxicity was accomplished by either inhibiting the Wnt pathway (comp.21, 22) or inhibiting the Smad pathway (comp.22), as well as docking against various targets connected to the K562 cell line.	[69]

### 6.4. Nanoparticles of Sesbania Species and Their Anticancer Use

*Sesbania*-based nanoparticles have shown unique properties due to the phytochemicals acting as reducing and stabilizing agents during nanoparticle synthesis. These nanoparticles have enhanced anticancer effects due to their ability to deliver bioactive compounds directly to cancer cells with improved bioavailability and targeted action.

#### 6.4.1. PEGylated Silver Nanoparticles of *Sesbania sesban*

Pandian et al. used *S. sesban* leaf extract to create silver nanoparticles, which were then coated in polyethyleneglycol (PEG) to increase their stability [70]. The physical properties of Ag and PEG-Ag nanoparticles were investigated using UV–vis spectrophotometer, XRD, FT-IR, EDX, and SEM. The physical characterization revealed that the Ag and PEG-Ag nanoparticles were spherical, with sizes ranging from 16 to 23 and 25 to 28 nm, respectively. The phytochemicals in the leaf extract act as reducing and stabilizing agents, leading to more stable nanoparticles. Also, the natural compounds in the extract may enhance the biocompatibility of the nanoparticles, making them safer for biomedical applications. In contrast, silver nanoparticles synthesized without *S. sesban* leaf extract may require additional chemical stabilizers, which can introduce toxicity and environmental concerns. By using *S. sesban* leaf extract, the synthesis of AgNPs not only leverages the plant’s natural properties for improved stability and biocompatibility but also aligns with sustainable and eco-friendly practices.

In addition, HeLa and macrophage (RAW 264.7) cells were used to investigate the immunomodulatory and anticancer properties of PEG-Ag NPs in vitro. The harmful effect of PEG-Ag NPs on HeLa and macrophage cells was examined using the MTT test. The findings indicated a 50% reduction in HeLa cell growth at a dosage of 1.5 μg mL^−1^, although no harmful effect was evident over 72 h. PEG-Ag NPs enhance macrophage growth at 1 μg mL^−1^. Additionally, they exhibit toxicity at 1.5 μg mL^−1^. Investigating PEG-Ag NPs’ stimulatory impact involved using cell sprouting and NBT experiments. The effects of PEG-Ag NPs on the innate immune system were then studied in vivo, employing mice as a model organism. The findings of both the in vitro and in vivo studies demonstrated the critical function PEG-Ag NPs play in immune cell activation and cancer growth prevention [71].

#### 6.4.2. Green Synthesized Silver Nanoparticles of *S. sesban*

Kuchekar et al. synthesized AgNPs and assessed the antimitotic activity of an aqueous extract of *S. sesban* seeds on growing Bengal gram seeds [72]. The results showed that the extract completely inhibited germination at doses greater than 500 μg/mL. The results also demonstrated that the extract had a deleterious reaction with Bengal gram seeds. These study substances showed various degrees of antimitotic activity. Testing for antimitotic action typically involves germination of Bengal gram seeds. With doses of 100, 500, and 1000 μg/mL, respectively, the AgNPs could prevent Bengal gram seeds from germinating, suggesting that they may have antimitotic activity. After the fifth day, growth was significantly inhibited by AgNPs, which demonstrated a strong antimitotic action at all doses. The cytotoxic medication works by interfering with cell division in dividing cells. Inhibiting mitosis in gram seed root tips is a sensitive and easy way to measure a drug’s cytotoxicity. Studies on the inhibition of mitosis in gram seedlings have revealed abnormalities in the production of cell plates and mitotic spindles, which may be connected to the arrest of cell division. Consequently, the research indicates that AgNPs’ cytotoxic effect may be able to stop cancer cells from going through the mitotic phase, which will be helpful in the treatment of cancer [72].

## 7. Discussion

Cancer remains one of the leading causes of death globally, prompting intensive efforts to develop both synthetic and natural therapeutic strategies. Natural treatments derived from plants, herbs, and other biological materials have gained significant attention due to their structural diversity and wide range of pharmacological properties. Among these, the *Sesbania* genus, particularly *S. grandiflora*, *S. sesban*, and *S. cannabina*, stands out as a promising source of bioactive compounds.

The *Sesbania* genus, a member of the *Fabaceae* family, consists of diverse species widely distributed across tropical and subtropical regions. Noted for its ecological versatility, *Sesbania* thrives in habitats ranging from riverbanks to swampy areas. Species like *S. sesban* and *S. grandiflora* are valued for their agricultural and industrial contributions, while others such as *S. punicea* are invasive and harmful to livestock. The conservation statuses of these species, as noted by the IUCN Red List, highlight the importance of monitoring their utilization to balance their ecological and economic significance.

Effective propagation techniques further emphasize the adaptability of *Sesbania* species. For instance, *S. sesban* is propagated through seeds, benefiting from scarification for improved germination, and thrives in saline and flood-prone soils. *S. grandiflora* is propagated using seeds or stem cuttings and adapts well to humid and hot climates but is sensitive to frost. *S. cannabina* requires boiling-water scarification for optimal seed germination and prefers consistently moist soils. These propagation methods underscore the importance of environmental management for successful cultivation and sustainable use of these plants.

The phytochemical diversity of *S. grandiflora* and *S. sesban* reveals significant variations depending on plant parts and extraction methods. S. grandiflora exhibits a broad spectrum of secondary metabolites, including alkaloids, flavonoids, tannins, saponins, terpenoids, and phenolic compounds, with the leaves being particularly rich in these compounds. Unique metabolites, such as sesbangrandiflorian A and B, demonstrate significant biological activities, including antiproliferative effects against cancer cell lines [38]. Compounds like vomifoliol, known for its anti-inflammatory and antioxidant properties, further highlight the therapeutic potential of *S. grandiflora*. Similarly, the roots and seeds contain nutritionally and medicinally valuable compounds, including isovestitol and tocopherols. *S. sesban* also contains a diverse range of phytochemicals, including unique sugars, sterols, triterpenes, and anthocyanins, depending on the extraction solvent used. Specialized compounds, such as oleanolic acid 3-β-D-glucuronide and cyanidin glucosides, underscore the importance of optimizing extraction techniques to maximize the yield of bioactive constituents.

To effectively harness the therapeutic potential of these species, future research should focus on systematically standardizing the extraction processes to ensure reproducibility and consistency in phytochemical profiles. In addition, robust preclinical studies are needed to evaluate the efficacy, pharmacokinetics, and safety profiles of these compounds in vivo. Developing formulations that enhance bioavailability and targeted delivery, such as encapsulation in nanoparticles, can further improve their therapeutic viability. Establishing a clear roadmap for clinical translation is also essential, involving steps such as toxicological assessments, regulatory compliance, and the design of clinical trials to evaluate their therapeutic efficacy in human populations. Addressing these gaps will facilitate the progression of *Sesbania*-based compounds from promising phytochemicals to clinically validated therapies.

The cytotoxic mechanism of *S. grandiflora* has been extensively studied. The F2 fraction induces apoptosis through both intrinsic and extrinsic pathways, evidenced by the activation of caspases 3, 8, and 9, cytochrome c release, and ROS generation. The dual role of ROS in inducing apoptosis and triggering autophagy further highlights the complex anticancer activity of *S. grandiflora* [73]. The downregulation of oncogenic pathways involving cyclin D1 and NF-κB reinforces its therapeutic potential. The presence of flavonoids and alkaloids also contributes to its cytotoxic effects, as well as its pharmacological safety, making *S. grandiflora* a strong candidate for anticancer drug development.

While *S. grandiflora* has been extensively studied, *S. cannabina* and *S. sesban* require further research to fully understand their potential. Preliminary findings indicate that galactomannan derivatives and 2-arylbenzofuran compounds from *S. cannabina* exhibit cytotoxic effects by upregulating caspase-12 levels [73]. Similarly, *S. sesban* inhibits several pathways involved in cancer progression, including the Wnt and Smad pathways, and shows potential in molecular docking studies on various cancer cell targets.

The application of nanotechnology in *Sesbania* research has also opened new avenues for enhancing anticancer efficacy. Nanoparticles synthesized from *Sesbania* species, such as cadmium oxide nanoparticles from S. grandiflora and silver or PEGylated silver nanoparticles from *S. sesban*, show promise in cancer therapy by preventing mitotic progression or activating immune responses to suppress tumor growth.

In conclusion, the *Sesbania* genus demonstrates immense potential as a source of novel anticancer agents. While the anticancer properties of S. grandiflora are well documented, further exploration of *S. sesban* and *S. cannabina* is warranted to uncover additional therapeutic opportunities. Advances in extraction techniques, pharmacological testing, and nanotechnology integration are crucial for realizing the full potential of these species in clinical applications. Despite the need for further research, *Sesbania* species offer a promising platform for the development of innovative anticancer drugs.

## 8. Future Aspects for Study

To fully unlock the therapeutic potential of *Sesbania* species, future research should prioritize underexplored areas and employ advanced methodologies. *S. cannabina*, in particular, warrants greater scientific attention. Investigating its secondary metabolites, such as phenolic compounds and alkaloids, through modern chromatographic techniques (e.g., HPLC, LC-MS, and GC-MS) could uncover novel bioactive compounds responsible for its anticancer properties. Additionally, systematic studies on other *Sesbania* species could help expand the repertoire of plant-based anticancer agents.

Understanding the molecular mechanisms underlying the anticancer effects of *Sesbania* species requires targeted biological studies to identify specific genes and proteins modulated during their activity. Proteomic and transcriptomic analyses could pinpoint overexpressed or downregulated pathways, elucidating how *Sesbania*-derived compounds disrupt cancer cell homeostasis. These insights would not only deepen scientific knowledge but also aid in designing more effective anticancer therapies.

The majority of current *Sesbania* research relies heavily on in vitro studies using cancer cell lines, which, while informative, provide limited insight into the complex interactions within living organisms. Future studies must include rigorous in vivo experiments to validate these findings, focusing on evaluating efficacy, pharmacokinetics, pharmacodynamics, and safety profiles of *Sesbania*-derived compounds. Advanced animal models mimicking human cancers could provide more relevant preclinical data. To bridge the gap between preclinical research and clinical application, well-designed clinical trials are essential. These trials should focus on determining the optimal dosing regimens, therapeutic windows, and long-term effects of *Sesbania*-based treatments. Collaborations between pharmaceutical scientists and clinical researchers will be critical to translating preclinical findings into real-world therapies.

Nanotechnology represents an exciting frontier for *Sesbania*-based cancer therapy. Nanoparticles synthesized from *Sesbania* species have demonstrated promising in vitro results, such as enhanced bioavailability, targeted drug delivery, and reduced toxicity. However, translating these benefits into clinical practice requires optimizing nanoparticle formulations, including surface modifications for improved specificity and stability. Detailed in vivo studies are essential to assess the pharmacokinetics, biodistribution, and therapeutic efficacy of these nanoparticles in complex biological systems. Furthermore, research should explore synergistic effects by combining *Sesbania*-derived nanoparticles with existing chemotherapeutics or immunotherapies. The clinical outlook for *Sesbania*-based therapies remains optimistic but requires addressing several challenges. Standardized protocols for plant cultivation, extraction, and compound isolation must be developed to ensure reproducibility and consistency in future studies. Regulatory guidelines for the safety and efficacy of *Sesbania*-derived compounds and nanoparticles should also be established to facilitate their transition into clinical use.

In conclusion, a multidisciplinary approach integrating advanced analytical techniques, molecular biology, nanotechnology, and clinical research is essential for realizing the full potential of *Sesbania*-based therapies. By addressing the outlined gaps, future research can significantly contribute to the development of innovative and effective anticancer treatments derived from *Sesbania* species.

## Figures and Tables

**Table 1:** Status of the selected species of *Sesbania* Adans according to the IUCN Red List of Threatened Species.

Species	IUCN Status	Last Assessed	Scope of Assessment	Year Published	Current Population Trend
*Sesbania sesban*	Least Concern (LC)	2 April 2019	Global	2020	Stable
*Sesbania grandiflora*	Data Deficient (DD)	28 February 2023	Global	2023	Unknown
*Sesbania cannabina*	Least Concern (LC)	21 July 2010	Global	2012	Stable

**Table 2:** Phytochemical compounds, extracted plant parts, and solvents used for extraction in *Sesbania grandiflora.* The percentages represent the relative concentration of each phytochemical compound within the extracted plant part, based on the solvent used, as reported in the referenced studies. These values are indicative and may vary depending on extraction methods and experimental conditions.

Plant Part	Solvent Fraction	Phytochemicals	References
Bark	Ethanol	Saponins (5%), tannins (3%), triterpenes (2%), and 2 arylbenzofurans (Sesbagrandiforian A and B)	[41,43]
	Ethyl acetate	Gallic acid (1.5%)	
Root	Ethanol	Alkaloids (4%), plant sterols/beta-sitosterol (2%), campesterol (1%), and stigmasterol (1%); glycosidic structures: glycosidic saponins (3%), bioflavonoids (2%), steroidal compounds (2%), and triterpenes (2%)	[43]
Seed	Ethanol	Leucocyanidin (2%), cyanidin (1.5%), saponins (3%), sesbanimide (1%)	[40]
	10% sodium hydroxide	Galactomannan (4%)	
	Acetone	Esterase B (1%) and esterase C (1%)	
	Hexane	Plant sterol/β-sitosterol (2%) and vitamin E (1%)	
Leaf	Ethanol	Alkaloids (3%), amino acids/proteins (4%), starch (5%), reducing and non-reducing sugars (4%), glycosides (3%), phenols (2%), saponins (3%), tannins (2%), terpenoids (2%), flavonoids such as quercetin and kaempferol (2%)	[36,37]
	Ethanol–water	Polyphenols (3%), carotenoids (2%), flavonoids (2%), and favanones (1.5%)	[42]
	50% and 70% aqueous ethanolic solution	Amino acids/proteins (4%), starch/carbohydrates (5%), calcium (1%), phenolic compounds (2%), and ascorbic acid (1%)	[43]
	Benzine	Starch/carbohydrates (5%), phenolic compounds (2%), amino acids/proteins (4%), steroids (2%), saponins (3%), tannins (2%), terpenoids (2%), flavonoids (2%), plant sterol/β-sitosterol (1%), and anthraquinone (1%)	[47]
	Ethyl acetate/methanol/chloroform	Polyphenols (3%), alkaloids (3%), flavonoids (2%), favanones (1.5%), amino acids/proteins (4%), starch/carbohydrates (5%), reducing sugar (2%), saponins (3%), and tannins (2%)	[13,16,42,47]
	Water	Alkaloids (3%), amino acids/proteins (4%), starch/carbohydrates (5%), glycosides/cyanogenic glycoside (1%), phenolic compounds (2%), and reducing sugars (2%)	[43]
Flower	Ethanol, 70% aqueous ethanolic solution	Kaempferol (1%), grandifloral (1%), amino acids/cystine (1%), isoleucine (1%), starch/carbohydrates (5%), glycosides (3%), steroids (2%), ascorbic acid (1%), tannins (2%), alkaloids (3%), flavonoids (2%)	[47,48]
	Methanol, ethyl acetate, water	Flavonoids (2%), tannins (2%), alkaloids (3%), anthraquinone (1%), glycosides (3%)	

**Table 3:** Phytochemical compounds, extracted plant parts, and solvents used for extraction in *Sesbania sesban*.

Plant Part	Solvent Fraction	Phytochemicals	References
Bark	Methanol, chloroform, petroleum ether	Alkaloids: 3%, carbohydrates: 5%, glycosides: 2%, phenols: 1.5%, saponins: 2%, plant sterols: 1%	[49,60]
	Ethanol ether (diethyl ether) chloroform	Alkaloids: 3%, carbohydrates: 5%, steroids: 2%, flavonoids: 1.5%, saponins: 2%, tannins: 1.5%	
	Water	Reducing sugars: 4%, sugar alcohols: 1.5%	
Root	Ethyl acetate and n-butanol-saturated extracts	Triterpenoids: 2%	[20]
Wood	Petroleum ether and chloroform ethyl acetate	Sterols: 2%, triterpenes: 1.5%, flavonoids: 1%	[52]
Leaves	Methanol, chloroform, and petroleum ether at 60–80°	Alkaloids: 3%, flavonoids: 2%, amino acids/proteins: 4%, fats: 2%, saponins: 2%, glycosides: 2%, plant sterols: 1%	[52]
	Water	Triterpenoids: 2%, carbohydrates: 5%, amino acids/proteins: 4%, tannins: 2%, saponins: 2%, glycosides: 2%, campesterol: 1%, cholesterol: 1%	[51,53]
Flower	Methanol and acidified methanol	Anthocyanins: 2%, phenols: 1.5%, flavonoids: 2%	[20,55]
Blossoms	Water	Glucosides: 2%	[50]
Pollen and dust tubes	Water	Keto-acids: 1.5%	[21]
Lignin	Water	Phenylpropanoid: 2%, flavonoids: 2%	[21,50]

## Data Availability

All the data for this research are available in the main manuscript.

## References

[1] McKenna D.J., Jones K., Hughes K., Tyler V.M. (2012). Botanical Medicines: The Desk Reference for Major Herbal Supplements.

[2] Li F.-S., Weng J.-K. (2017). Demystifying traditional herbal medicine with modern approach. Nat. Plants.

[3] Joshi S.G. (2000). Medicinal Plants.

[4] Sreelatha S., Padma P., Umasankari E. (2011). Evaluation of anticancer activity of ethanol extract of *Sesbania grandiflora* (Agati Sesban) against Ehrlich ascites carcinoma in Swiss albino mice. J. Ethnopharmacol..

[5] Naher U.A., Choudhury A.T., Biswas J.C., Panhwar Q.A., Kennedy I.R. (2020). Prospects of using leguminous green manuring crop Sesbania rostrata for supplementing fertilizer nitrogen in rice production and control of environmental pollution. J. Plant Nutr..

[6] Wachiebene S. (2021). Assessment of Feed Resources for Ruminant Production in Northern Region of Ghana.

[7] Bunma S., Balslev H. (2019). A review of the economic botany of Sesbania (Leguminosae). Bot. Rev..

[8] Zainab N., Khan A.A., Azeem M.A., Ali B., Wang T., Shi F., Alghanem S.M., Hussain Munis M.F., Hashem M., Amna (2021). PGPR-mediated plant growth attributes and metal extraction ability of *Sesbania sesban* L. in industrially contaminated soils. Agronomy.

[9] Royal Botanic Gardens, & Kew and Missouri Botanical Garden. http://www.theplantlist.org/browse/A/Leguminosae/Sesbania/.

[10] Farruggia F.T. (2009). Phylogenetic and Monographic Studies of the Pantropical Genus Sesbania adanson (Leguminosae).

[11] Compendium C.I.S. (2022). CAB International: Wallingford, UK. https://www.cabi.org/tag/isc/.

[12] Vijayakumar P., Singaravadivelan A., Senthilkumar D., Vasanthakumar T., Ramachandran M. (2021). Effect of *Sesbania grandiflora* (*Agati*) Supplementation on Weight Gain of Crossbred Jersey Heifer Calves. Int. J. Econ. Plants.

[13] Arfan N., Julie A., Mohiuddin A., Khan S., Labu Z. (2016). Medicinal properties of the sesbania grandiflora leaves. Ibnosina J. Med. Biomed. Sci..

[14] Ansil P., Soumya S., Shafna S. (2022). Sesbania grandiflora: A Potential Source of Phytopharmaceuticals.

[15] Balan A.P., Udayan P., Predeep S. (2023). Palynotaxonomical Studies on Selected Indian Endemic Legumes.

[16] Momin R., Kadam V. (2011). Biochemical analysis of leaves of some medicinal plants of genus Sesbania. J. Ecobiotechnol..

[17] Pandian A., Sivalingam A.M., Lakshmanan G., Semwal R., Selvakumari J., Semwal D. (2023). Phytochemical analysis and antioxidant activity of the leaf extract of *Sesbania grandiflora* (L.) Poiret. Curr. Med. Drug Res..

[18] Heuzé V., Tran G., Bastianelli D., Lebas F. (2015). Sesban (*Sesbania sesban*). NRA, CIRAD, AFZ and FAO. https://www.feedipedia.org/node/253.

[19] Saptarshi S., Ghosh A.K. (2017). Pharmacological effects of *Sesbania sesban* Linn: An overview. PharmaTutor.

[20] Goswami S., Mishra K., Singh R.P., Singh P., Singh P. (2016). *Sesbania sesban*, a plant with diverse therapeutic benefits: An overview. J Pharma. Res. Edu..

[21] Gomase P.V. (2012). *Sesbania sesban* Linn: A review on its ethnobotany, phytochemical and pharmacological profile. Asian J. Biomed. Pharm. Sci..

[22] Zuanny D.C., Vilela B., Moonlight P.W., Särkinen T.E., Cardoso D. (2024). expowo: An R package for mining global plant diversity and distribution data. Appl. Plant Sci..

[23] Lewis G.P., Schrire B., Mackinder B., Lock M. (2005). Legumes of the World.

[24] POWO Plants of The World Online. https://powo.science.kew.org.

[25] Betts J., Young R.P., Hilton-Taylor C., Hoffmann M., Rodríguez J.P., Stuart S.N., Milner-Gulland E. (2020). A framework for evaluating the impact of the IUCN Red List of threatened species. Conserv. Biol..

[26] Ghogue J.-P. *Sesbania sesban*. The IUCN Red List of Threatened Species. https://www.iucnredlist.org/species/185456/13560631.

[27] IUCN The IUCN Red List of Threatened Species. Version 2023-1. https://www.iucnredlist.org/species/168726/20141760.

[28] Chong K.Y., Koh C., Low Y.W., Lua H.K., Mustaqim W., Sam Y.Y., Yee W. (2023). Crudia wrayi. The IUCN Red List of Threatened Species 2023: E.T224976135A224976137.

[29] Oduol P.A., Akunda E. (1988). Vegetative Propagation of Sesbania sesban by Cuttings.

[30] Orwa C. (2009). Agroforestree Database: A Tree Reference and Selection Guide, Version 4.0. https://www.cifor-icraf.org/.

[31] Karmakar P., Singh V., Yadava R., Singh B., Singh R., Kushwaha M. Agathi [*Sesbania grandiflora* L. (Agast)]: Current status of production, protection and genetic improvement: IIVR-Regional Research Station, Sargatia, Kushinagar, Uttar Pradesh-274406. Proceedings of the National Symposium on Vegetable Legumes for Soil and Human Health.

[32] Iqbal N., Manalil S., Chauhan B.S., Adkins S.W. (2019). Germination biology of sesbania (*Sesbania cannabina*): An emerging weed in the Australian cotton agro-environment. Weed Sci..

[33] Anulika N.P., Ignatius E.O., Raymond E.S., Osasere O.-I., Abiola A.H. (2016). The chemistry of natural product: Plant secondary metabolites. Int. J. Technol. Enhanc. Emerg. Eng. Res.

[34] Velu G., Palanichamy V., Rajan A.P., Roopan S.M., Madhumitha G. (2018). Phytochemical and pharmacological importance of plant secondary metabolites in modern medicine: In Bioorganic Phase in Natural Food: An Overview. Bioorganic Phase in Natural Food: An Overview.

[35] Patil P., Shah N. (2022). *Sesbania grandiflora* (L.) Pers.(Agati): Its Ethnobotanical Knowledge, Phytochemical Studies, Pharmacological Aspects, and Future Prospects. TMR Integr. Med..

[36] Hussain A.Z., Ignatius A. (2010). GC-MS Analysis and Antimicrobial Activity of Acalypha indica Linn.

[37] Emitaro W.O., Musyimi D.M., Opande G.T., Odiambo G. (2020). Phytochemical and antimicrobial properties of leaf extracts of *Calliandra calothyrsus*, *Leucaena diversifolia* and *Sesbania sesban*. Bact. Emp..

[38] Deepthi K., Renjith P., Habeeb Rahman K., Chandramohanakumar N. (2024). A comprehensive review of *Sesbania grandiflora* (L.) Pers: Traditional uses, phytochemistry and pharmacological properties. Vegetos.

[39] Padmalochana K., Rajan M.D. (2014). Antimicrobial activity of Aqueous, Ethanol and Acetone extracts of *Sesbania grandiflora* leaves and its phytochemical characterization. Int. J. Pharma Sci. Res..

[40] Vinothini K., Devi M.S., Shalini V., Sekar S., Semwal R.B., Arjun P., Semwal D.K. (2017). In vitro micropropagation, total phenolic content and comparative antioxidant activity of different extracts of *Sesbania grandiflora* (L.) Pers. Curr. Sci..

[41] Noviany N., Samadi A., Yuliyan N., Hadi S., Aziz M., Purwitasari N., Mohamad S., Ismail N.N., Gable K.P., Mahmud T. (2020). Structure characterization and biological activity of 2-arylbenzofurans from an Indonesian plant, *Sesbania grandiflora* (L.) Pers. Phytochem. Lett..

[42] Ajitha B., Reddy Y.A.K., Rajesh K., Reddy P.S. (2016). *Sesbania grandiflora* leaf extract assisted green synthesis of silver nanoparticles: Antimicrobial activity. Mater. Today Proc..

[43] Arthanari S., Periyasamy P. (2020). Phenolic composition, antioxidant and anti-fibrotic effects of *Sesbania grandiflora* L. (Agastya)—An edible medicinal plant. AYU (Int. Q. J. Res. Ayurveda).

[44] Siddhuraju P., Abirami A., Nagarani G., Sangeethapriya M. (2014). Antioxidant capacity and total phenolic content of aqueous acetone and ethanol extract of edible parts of *Moringa oleifera* and *Sesbania grandiflora*. Int. J. Biol. Biomol. Agric. Food Biotechnol. Eng..

[45] Chandralekha B., Kailash G., Anuprita J. (2022). Assessment of nutritional and phytochemical properties of *Sesbania grandiflora* flower and leaves. Pharma Innov. J..

[46] Mohiuddin A.K. (2019). Medicinal and therapeutic values of *Sesbania grandiflora*. J. Pharm. Sci. Exp. Pharmacol..

[47] Naqi M. (2014). Comparative of Phytochemical and Antimicrobial of *Sesbania grandiflora* Leaves Extract. Med. J. Babylon.

[48] Saifudin A., Forentin A.M., Fadhilah A., Tirtodiharjo K., Melani W.D., Widyasari D., Saroso T.A. (2016). Bioprospecting for anti-Streptococcus mutans: The activity of 10% *Sesbania grandiflora* flower extract comparable to erythromycin. Asian Pac. J. Trop. Biomed..

[49] Brahim Mahamat O., Younes S., Otchom B.B., Franzel S., Ouchar Mahamat Hidjazi A.-D., Soumaya E.i. (2024). A Review on Medicinal and Ethnomedicinal Uses, Biological Features, and Phytochemical Constituents of *Sesbania sesban* L. Merr., A Nitrogen-Fixing Plant Native to the Republic of Chad. Sci. World J..

[50] Rageeb M., Usman M., Patil S.B., Patil S.S., Patil R.S. (2013). *Sesbania sesban* Linn.: An overview. Int. J. Pharm. Life Sci..

[51] Soren A.D., Chen R.P., Yadav A.K. (2021). In vitro and in vivo anthelmintic study of *Sesbania sesban* var. bicolor, a traditionally used medicinal plant of Santhal tribe in Assam, India. J. Parasit. Dis..

[52] Yadav A.K., Gogoi S., Vijaya (2018). In vitro and in vivo anthelmintic efficacy of two pentacyclic triterpenoids, ursolic acid and betulinic acid against mice pinworm, *Syphacia obvelata*. J. Parasit. Dis..

[53] Santos F.O., Cerqueira A.P.M., Branco A., Batatinha M.J.M., Botura M.B. (2019). Anthelmintic activity of plants against gastrointestinal nematodes of goats: A review. Parasitology.

[54] Marappan S., Manickam K., Devi P.S. (2012). Bioactive compounds in *Sesbania sesban* flower and its Antioxidant and Antimicrobial activity. J. Pharm. Res..

[55] Chaiyasut C., Sivamaruthi B.S., Pengkumsri N., Sirilun S., Peerajan S., Chaiyasut K., Kesika P. (2016). Anthocyanin profile and its antioxidant activity of widely used fruits, vegetables, and flowers in Thailand. Asian J. Pharm. Clin. Res..

[56] Abdelgawad S.M., Hetta M.H., Ibrahim M.A., Fawzy G.A., El-Askary H.I., Ross S.A. (2023). Holistic Overview of the Phytoconstituents and Pharmacological Activities of Egyptian Riverhemp [*Sesbania sesban* (L.) Merr.]: A Review. Nat. Prod. Commun..

[57] Boesten D.M., den Hartog G.J., de Cock P., Bosscher D., Bonnema A., Bast A. (2015). Health effects of erythritol. Nutrafoods.

[58] Fouillaud M.C.Y., Venkatachalam M., Grondin I., Dufossé L., Nollet L.M.L., Gutiérrez-Uribe J.A. (2018). Anthraquinones. Phenolic Compounds in Food Charac-terization and Analysis.

[59] Ahmed A., Labu Z., Dey S., Hira A., Howlader M., Hossain M., Roy J. (2013). Phytochemical screening, antibacterial and cytotoxic activity of different fractions of *Xylocarpus mekongensis* Bark. Ibnosina J. Med. Biomed. Sci..

[60] Laladhas K.P., Cheriyan V.T., Puliappadamba V.T., Bava S.V., Unnithan R.G., Vijayammal P.L., Anto R.J. (2010). A novel protein fraction from *Sesbania grandiflora* shows potential anticancer and chemopreventive efficacy, in vitro and in vivo. J. Cell. Mol. Med..

[61] Joselin A.P. (2006). The Role of the Apoptosis and Splicing Associated Protein Acinus During Apoptotic Nuclear Changes. Ph.D. Thesis.

[62] Roy R., Kumar D., Chakraborty B., Chowdhury C., Das P. (2013). Apoptotic and autophagic effects of *Sesbania grandiflora* flowers in human leukemic cells. PLoS ONE.

[63] Ponnanikajamideen M., Nagalingam M., Vanaja M., Malarkodi C., Rajeshkumar S. (2015). Anticancer activity of different solvent extracts of *Sesbania grandiflora* against neuroblastima (imr-32) and colon (ht-29) cell lines. Eur. J. Biomed. Pharm. Sci.

[64] Pajaniradje S., Mohankumar K., Pamidimukkala R., Subramanian S., Rajagopalan R. (2014). Antiproliferative and apoptotic effects of *Sesbania grandiflora* leaves in human cancer cells. BioMed Res. Int..

[65] Padmalochana K., Rajan M.D. (2015). In-vitro anticancer activity of different extracts of *Sesbania grandiflora* against HEP2 cell lines. World J. Pharm. Sci..

[66] Zhou M., Yang L., Yang S., Zhao F., Xu L., Yong Q. (2018). Isolation, characterization and in vitro anticancer activity of an aqueous galactomannan from the seed of Sesbania cannabina. Int. J. Biol. Macromol..

[67] Mehta N., Rao P., Saini R. (2019). Evaluation of antioxidant and anticancer potential of Sesbania aculeata-a multipurpose legume crop. Ann. Food Sci. Technol..

[68] Fu X.-J., Yi J.-L., Yang J.-Y., Lin X.-Q., Huang W.-H., Zhou X.-M. (2021). Bioactive 2-arylbenzofurans derivatives from Sesbania cannabina. Phytochem. Lett..

[69] Abdelgawad S.M., Hetta M.H., Ibrahim M.A., Balachandran P., Zhang J., Wang M., Eldehna W.M., Fawzy G.A., El-Askary H.I., Ross S.A. (2022). Phytochemical investigation of Egyptian Riverhemp: A potential source of antileukemic metabolites. J. Chem..

[70] Dianhar H., Syah Y.M., Mujahidin D., Hakim E.H., Juliawaty L.D. A flavone derivative from *Sesbania sesban* leaves and its cytotoxicity against murine leukemia P-388 cells. Proceedings of the AIP Conference Proceedings.

[71] Pandian S.R.K., Anjanei D., Raja N.L., Sundar K. (2018). PEGylated silver nanoparticles from Sesbania aegyptiaca exhibit immunomodulatory and anti-cancer activity. Mater. Res. Express.

[72] Kuchekar A., Desai R., Gawade A. (2022). Antimitotic Activity of Green Synthesized Silver Nanoparticles by Seed Extract of *Sesbania Sesban*. Mater. Int..

[73] Chen Y., Gibson S.B. (2008). Is mitochondrial generation of reactive oxygen species a trigger for autophagy?. Autophagy.

